# Olfactory Deficits in the Freezing of Gait Phenotype of Parkinson's Disease

**DOI:** 10.3389/fneur.2021.656379

**Published:** 2021-08-12

**Authors:** Aliyah Glover, Lakshmi Pillai, Rohit Dhall, Tuhin Virmani

**Affiliations:** ^1^Department of Neurology, University of Arkansas for Medical Sciences, Little Rock, AR, United States; ^2^Center for Translational Neuroscience, University of Arkansas for Medical Sciences, Little Rock, AR, United States

**Keywords:** freezing of gait, Parkinson's disease, olfaction, Anosmia, UPSIT

## Abstract

**Background:** Olfactory dysfunction often occurs before motor onset in Parkinson's disease (PD) and can be detected with the University of Pennsylvania Smell Identification Test (UPSIT). Based on the Braak hypothesis, the olfactory bulb is one of two sites where disease pathology may start and spread to deeper brain structures.

**Objective:** To evaluate whether a specific pattern of odorant identification on the UPSIT discriminated Parkinson's disease patients with and without freezing of gait.

**Methods:** One hundred and twenty four consecutive participants (33 controls, 31 non-freezers, and 60 freezers) were administered the UPSIT. Using the chi-square test, each odorant on the UPSIT was ranked based on the differential ability of freezers and non-freezers to identify them correctly. Using predictive statistics and confusion matrices, the best combination of odorants and a cut-off score was determined.

**Results:** Freezers had a shift toward a more severe hyposmia classification based on age and sex based normative values. The correct identification of nine odors (bubblegum, chocolate, smoke, wintergreen, paint thinner, orange, strawberry, grass, and peanut) was significantly worse in freezers compared to non-freezers. Correctly identifying ≤ 2 out of 3-odorants (bubblegum, chocolate, and smoke) had a 77% sensitivity and 61% specificity for categorizing freezers. The 3-odorant score was not correlated with disease duration, motor or total UPDRS scores, MoCA scores or age at testing. The predictive statistics were similar when sexes were separately categorized.

**Conclusions:** A 3-odorant score helped categorize freezers and non-freezers with similar sensitivity and specificity to short odorant Parkinson's disease identification batteries.

## Introduction

Olfactory dysfunction is reported in 46–98% of patients with Parkinson's Disease (PD) (1–3). Olfactory neurons are among the first to display Lewy body pathology (4) and clinical hyposmia often occurs years before motor symptoms manifest (5, 6). The 40-item University of Pennsylvania Smell Identification Test (UPSIT) is an effective instrument to detect olfactory dysfunction in PD (7–9), however the time and cost to administer it has limited clinical use. Shorter tests derived from the UPSIT, have been shown to have similar sensitivity and specificity to the extended version (10–13), including tests that consider cultural variation in exposure to different odorants such as the brief smell identification test (B-SIT) (10).

Whether the severity of smell deficit in PD patients correlates with disease duration or severity is still a matter of debate, with some studies reporting worsened olfactory deficit with disease severity (14–16), and others reporting no relationship (17–20). Different tests used in these studies could partly account for the heterogeneous results. One such study found participants with B-SIT scores lower than the 20th percentile had higher freezing of gait questionnaire (FOG-Q) scores (11). Longitudinal evaluation of olfactory deficits in smaller cohorts have also reported unchanged (19) or worsening olfaction over time (21). Lower dopamine transporter levels have been correlated with lower UPSIT scores (15, 22, 23).

There is also growing evidence to suggest that PD is a heterogeneous condition, with different subgroups more susceptible to earlier (vs. later) development of both non-motor and motor manifestations of the disease, including freezing of gait (24). As the Braak hypothesis suggests that PD pathology begins in the olfactory bulb or intestine (4), different disease subtypes could be dependent on subtle differences in this early pathology. We hypothesized that PD patients with freezing of gait (freezers) would have a differential pattern of dysfunction in odorant identification than PD patients without freezing of gait (non-freezers). To test this we administered the UPSIT to a population of PD patients and aging controls and analyzed individual odorant identification in these different groups to identify a subset of odorants that most accurately categorized PD freezers and non-freezers.

## Methods

People with idiopathic PD, based upon the UK Brain Bank Criteria (25), and age-matched controls, were recruited from the Movement Disorders Clinic at the University of Arkansas for Medical Sciences (UAMS) after approval from the Institutional Review Board (IRB) and in compliance with the Helsinki guidelines for research involving human participants (UAMS IRB# 203234). All participants provided written consent before participating. This cohort was prospectively enrolled in a longitudinal gait study which had enrollment exclusion criteria of >1 fall/day, a Montreal cognitive assessment (MoCA) score <10, and dopamine-receptor antagonist use in the prior year. Either the participant or a power of attorney (POA), if indicated, provided written informed consent. If participants MoCA scores dropped below 10 at any time during their active participation in the study, participants were asked to re-consent before they could continue participation with a POA/spouse serving as a cosigner. Exposures that could have affected odorant identification were not part of the longitudinal study's exclusion criteria, but were documented in a health history questionnaire that included past medical history, surgical history, medications and exposures. Clinical history was updated at each visit. Smoking history of participants was additionally documented as part of the UPSIT administration questionnaire. The complete grouped medical and surgical history of all subjects is provided in Supplementary Table 1.

The first 124 consecutive participants to complete the UPSIT were included in the analysis for this report. On the day of their UPSIT, all participants were administered a Unified Parkinson's Disease Rating Scale (UPDRS) score (26), a Hoehn and Yahr staging score (27), the FOG-Q (28), and a MoCA (29). All study assessments were performed in the ON-medication state.

Participants were classified as freezers on the day of their UPSIT, if they had a score of ≥1 on UPDRS item 14 (equivalent to FOG-Q item 3 > 1) after a demonstration of gait freezing, or if they had witnessed gait freezing on exam by a movement disorders specialist (T.V.), corresponding to probable or definite freezers based on prior criteria (30).

The 40-item scratch-and-sniff UPSIT (8) was administered (Smell Identification Test^TM^, Sensonics, Inc., NJ) with care taken to avoid strong odorants in the test taking environment (such as coffee), that could influence performance. If inability to smell an odorant was reported, in order to ensure that it was not secondary to a faulty test, the sample was re-scratched one additional time and the examiner ensured that they could smell the odorant. Participants were required to select an answer from the multiple choices on the test before moving to the next odorant.

SPSS v24 (IBM) was used for statistical analysis. Normality was assessed using the Shapiro–Wilk test, and statistical significance calculated using a one-way ANOVA (parametric) or the Kruskal–Wallis test (non-parametric data) with a *post-hoc* Bonferroni correction for multiple group comparisons. Pearson's correlation coefficients were calculated for clinical assessment scores compared to the final 3-odorant scores. The Kolmogorov–Smirnov *Z*-test was used to compare distributions. A chi-square test was used to determine the significance of group differences for nominal variables.

Predictive statistics were performed to determine the pattern and types of odors best discriminating non-freezers from freezers (9). UPSIT items were ranked based on the results of individual chi-square tests between the non-freezer and freezer groups. The UPSIT items were then iteratively combined in order of statistical significance, and confusion matrices for different cut-offs for 3-odorants up to 8-odorants determined. The sensitivity, specificity, positive (PPV), and negative predictive values (NPV) were calculated from the confusion matrices, to determine the group of odorants and score, that best categorized freezers and non-freezers.

## Results

**Figure 1:**
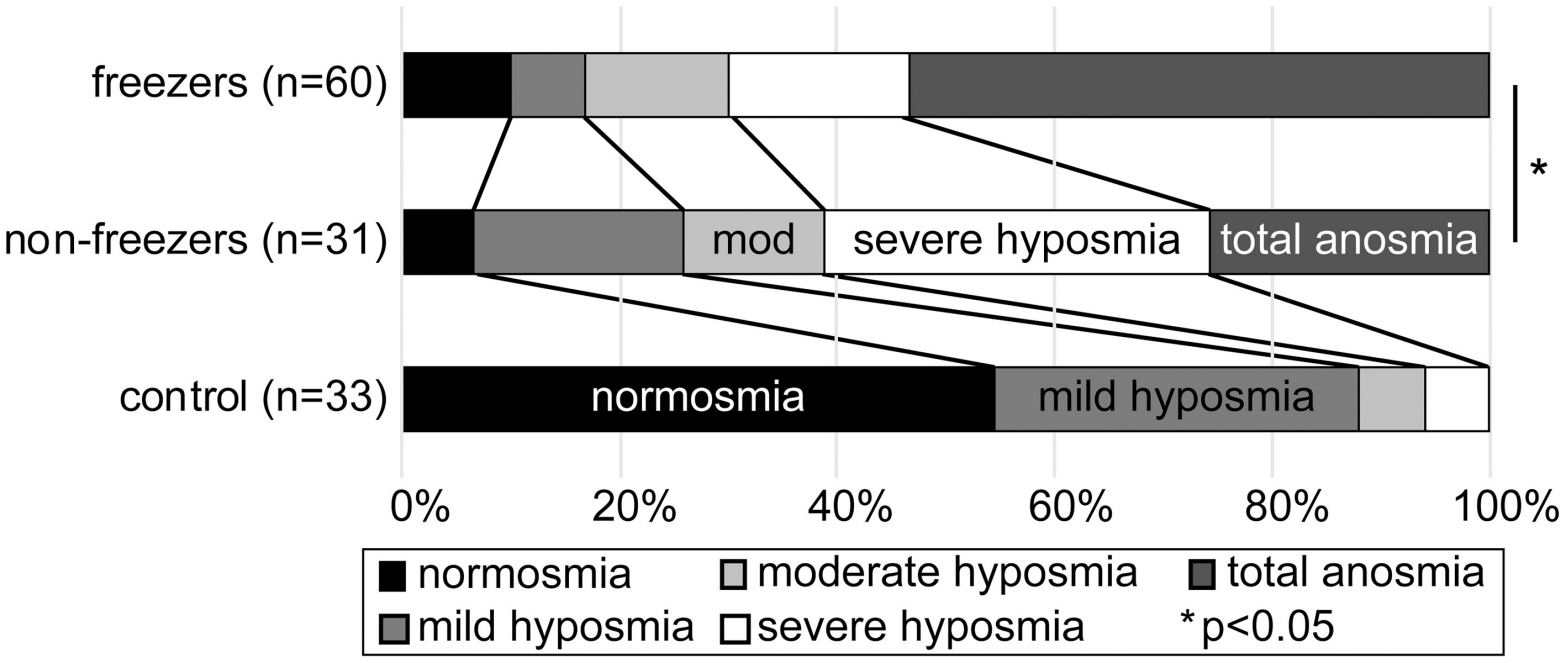
Stacked bar graph showing percentage severity of hyposmia or anosmia in each group, on the University of Pennsylvania Smell Identification test, based on age and sex controlled established normative values. Healthy controls (bottom bar), PD without freezing (middle bar), and PD with freezing (upper bar) are shown. PD freezers were significantly more likely to have an anosmic phenotype than non-freezers.

The first 124 participants in our longitudinal study to undergo an UPSIT (33 controls, 31 non-freezers and 60 freezers) were included in the analysis, and their demographics are shown in Table 1. Utilizing the age and sex based normative score classification for the UPSIT provided with the test, freezers had a significant shift toward more severe phenotypes compared to non-freezers (Figure 1) using a chi-square test (*p* = 0.041), and was approaching significance using a test for distributions (Kolmogorov–Smirnov *Z*-score 1.244, *p* = 0.09).

**Table 1:** Participant demographics and potential olfactory modulators.

	**Controls (*n* = 33)**	**Non-freezers (*n* = 31)**	**Freezers (*n* = 60)**
Sex, No. M/F^#^	12/21	20/11	34/26
Age (years) ^+^	65.8 ± 7.6	70.7 ± 12.6	68.0 ± 8.4
MoCA score^@^	27.6 ± 2.1	26.3 ± 2.6	23.8 ± 4.9
Disease duration (years)^@^	–	6.2 ± 3.9	11.0 ± 6.3^***^
Hoehn and Yahr score^@^	–	1.7 ± 0.5	2.6 ± 0.8^**^
FOG-Q score^@^	0.3 ± 0.9	1.3 ± 1.2	10.2 ± 5.2^***^
UPDRS Part III score (motor)^@^	3.3 ± 2.8	12.4 ± 6.4	22.2 ± 10.3^**^
Total UPDRS score^@^	5.8 ± 4.0	21.0 ± 8.4	40.8 ± 16.7^***^
UPSIT score^@^	33.8 ± 4.1	23.5 ± 7.5	19.8 ± 8.3
**Potential olfactory modulators [No. (%)]**			
Smoking history^#^	12 (36%)	12 (39%)	25 (42%)
Use of nasal sprays^#^	3 (9%)	3 (10%)	4 (7%)
Seasonal allergies^#^	4 (6%)	2 (6%)	2 (3%)
History of asthma/COPD^#^	2 (6%)	0 (0%)	6 (10%)
History of head trauma^#^	1 (3%)	1 (3%)	6 (10%)
History of deviated septum or nasal surgery^#^	1 (3%)	0 (0%)	1 (2%)
Diabetes^#^	2 (6%)	1 (3%)	3 (5%)
Alcohol abuse/thiamine deficiency^#^	0 (0%)	0 (0%)	0 (0%)
Chemical exposures (including pesticides, welding, other occupational)^#^	1 (3%)	5 (16%)	8 (13%)
**Medications (of 71 reported, excluding levodopa)** ^**a**^			
Levothyroxine^#^	3 (9%)	6 (19%)	6 (12%)
Atorvastatin^#^	3 (9%)	4 (13%)	4 (7%)
Amlodipine^#^	1 (3%)	5 (16%)^*^	1 (2%)
Pravastatin^#^	0 (0%)	3 (10%)	4 (7%)
Corticosteroids (oral or inhaled)^#^	2 (6%)	0 (0%)	2 (3%)
Sildenafil^#^	0 (0%)	1 (3%)	1 (2%)
Sumatriptan^#^	0 (0%)	1 (3%)	0 (0%)
Cholestyramine^#^	0 (0%)	0 (0%)	1 (2%)

*Statistical value reported with post-hoc Bonferonni correction.*

* *p < 0.05,*

** *p < 0.01,*

*** *p < 0.001. Tests used were:*

# *chi-square, ^+^One-Way ANOVA, @Kruskal–Wallis,*

*^a^(31)*.

Nine of the forty odors (bubblegum, chocolate, smoke, wintergreen, paint thinner, orange, strawberry, grass, and peanut) were more frequently incorrectly identified in freezers compared to non-freezers (chi-square; Table 2). In order to determine the best combination of odors that could categorize freezers and non-freezers, we started with the 3 odors with highest individual chi-square values (bubblegum, chocolate, and smoke), and calculated the sensitivity, specificity, PPV and NPV from confusion matrices for different cut-off values (Table 3). Successive odors were added, and statistics re-calculated for each combination (Table 3). The 3-odorants (bubblegum, chocolate, and smoke), with correct identification of ≤ 2 odors, was as good at categorizing freezers and non-freezers (82% sensitivity, 52% specificity, 77% PPV, and 59% NPV) as 6-odorants (bubblegum, chocolate, smoke, wintergreen, paint thinner, and orange) with correct identification of ≤ 4 odors (77% sensitivity, 61% specificity, 79% PPV, and 58% NPV). The same 3-odorants (bubblegum, chocolate, and smoke), with a cut off ≤ 2 odors identified, also had a 70% sensitivity, 67% specificity, 85% PPV, and 45% NPV for categorizing our PD participants from our controls.

**Table 2:** Individual odorant categorization statistics for freezers vs. non-freezers.

**UPSIT** **Item #**	**Odorant**	**Chi-square** ***p*-value**	**Chi-square value**	**Non-freezers number incorrect**	**Freezers number incorrect**	**Differential number of freezers with incorrect answers**	**Differential percent of freezers with incorrect answers**	**Sensitivity**	**Specificity**	**PPV**	**NPV**
2	Bubblegum	**0.001**	13.052	10	43	33	39%	0.72	0.68	**0.81**	0.55
19	Chocolate^a^	**0.022**	5.264	2	16	14	20%	0.27	**0.94**	**0.89**	0.40
33	Smoke^a, c, d, e^	**0.033**	4.53	6	25	19	22%	0.42	**0.81**	**0.81**	0.42
29	Wintergreen^b, g^	**0.038**	4.299	8	29	21	23%	0.48	0.74	**0.78**	0.43
31	Paint thinner^a, b^	**0.038**	4.299	8	29	21	23%	0.48	0.74	**0.78**	0.43
28	Orange^h^	**0.039**	4.251	9	31	22	23%	0.52	0.71	**0.78**	0.43
17	Strawberry^b, c^	**0.039**	4.241	10	33	23	23%	0.55	0.68	**0.77**	0.44
32	Grass^b^	**0.039**	4.241	10	33	23	23%	0.55	0.68	**0.77**	0.44
40	Peanut	**0.047**	3.942	6	24	18	21%	0.40	**0.81**	**0.80**	0.41
11	Onion^a^	0.064	3.437	2	13	11	15%	0.22	**0.94**	**0.87**	0.38
39	Rose^a, b, c, h, i, k^	0.073	3.214	13	37	24	20%	0.62	0.58	0.74	0.44
23	Peach	0.105	2.634	9	28	19	18%	0.47	0.71	**0.76**	0.41
4	Cherry^c^	0.142	2.157	10	29	19	16%	0.48	0.68	0.74	0.40
21	Lilac^c, e^	0.142	2.157	10	29	19	16%	0.48	0.68	0.74	0.40
1	Pizza^b, g^	0.187	1.745	21	32	11	−14%	0.53	0.32	0.60	0.26
8	Clove^b, c, h, k^	0.212	1.555	6	19	13	12%	0.32	**0.81**	**0.76**	0.38
16	Gasoline^a, d^	0.232	1.427	14	35	21	13%	0.58	0.55	0.71	0.40
5	Motor Oil	0.237	1.396	18	27	9	−13%	0.45	0.42	0.60	0.28
14	Cheddar Cheese	0.241	1.375	21	33	12	−13%	0.55	0.32	0.61	0.27
15	Cinnamon^a, d, h, i^	0.241	1.377	12	31	19	13%	0.52	0.61	0.72	0.40
13	Licorice^b, f, h, i^	0.269	1.222	17	40	23	12%	0.67	0.45	0.70	0.41
30	Watermelon	0.29	1.119	16	24	8	−12%	0.40	0.48	0.60	0.29
25	Dill Pickle^f^	0.302	1.064	22	36	14	−11%	0.60	0.29	0.62	0.27
34	Pine	0.306	1.048	12	30	18	11%	0.50	0.61	0.71	0.39
36	Lemon^a, b, c, e, h^	0.366	0.817	15	35	20	10%	0.58	0.52	0.70	0.39
3	Menthol^c^	0.372	0.796	8	21	13	9%	0.35	0.74	0.72	0.37
38	Natural gas^c^	0.382	0.765	12	29	17	10%	0.48	0.61	0.71	0.38
18	Cedar	0.453	0.564	15	34	19	8%	0.57	0.52	0.69	0.38
12	Fruit Punch	0.517	0.42	18	39	21	7%	0.65	0.42	0.68	0.38
9	Leather^c, h^	0.545	0.367	6	15	9	6%	0.25	**0.81**	0.71	0.36
26	Pineapple^a, c, d, h, i^	0.562	0.337	13	29	16	6%	0.48	0.58	0.69	0.37
37	Soap^a, c^	0.655	0.2	15	32	17	5%	0.53	0.52	0.68	0.36
24	Root beer	0.662	0.192	17	30	13	−5%	0.50	0.45	0.64	0.32
35	Grape	0.75	0.102	14	25	11	−3%	0.42	0.55	0.64	0.33
10	Coconut^b^	0.759	0.094	15	27	12	−3%	0.45	0.52	0.64	0.33
6	Mint^h, i, j^	0.905	0.014	12	24	12	1%	0.40	0.61	0.67	0.35
20	Gingerbread	0.918	0.011	10	20	10	1%	0.33	0.68	0.67	0.34
27	Lime^b^	0.921	0.01	24	47	23	1%	0.78	0.23	0.66	0.35
22	Turpentine^a, h^	0.972	0.001	19	37	18	0%	0.62	0.39	0.66	0.34
7	Banana^a, b, d, f, h, i^	0.98	0.001	18	35	17	0%	0.58	0.42	0.66	0.34

*^a^B-SIT, ^b^B-SIT B (PD), ^c^B-SIT A, ^d^Double, ^e^Pocket Smell Test, ^f^Bohnen, ^g^Hawkes, ^h^Odor pen-based Sniffin' Sticks, ^i^Mahlknect, ^j^Casjens, ^k^Hummel. Bolded items in chi-square and p-value column highlight significance (p < 0.05). Bolded items in sensitivity, specificity, PPV, and NPV columns highlight values >0.75*.

**Table 3:** Binary classification results for odorant combinations for freezers vs. non-freezers.

**Number correct**	**Sensitivity**	**Specificity**	**PPV**	**NPV**
1 out of 3	0.48	0.90	0.91	0.47
**2 out of 3**	**0.82**	**0.52**	**0.77**	**0.59**
3 out of 3	1.00	0.00	0.66	0.00
1 out of 4	0.33	0.94	0.91	0.42
2 out of 4	0.62	0.90	0.93	0.55
3 out of 4	0.85	0.32	0.71	0.53
2 out of 5	0.47	0.90	0.90	0.47
3 out of 5	0.68	0.71	0.82	0.54
4 out of 5	0.88	0.32	0.72	0.59
2 out of 6	0.42	0.97	0.96	0.46
3 out of 6	0.57	0.84	0.87	0.50
4 out of 6	0.77	0.61	0.79	0.58
5 out of 6	0.93	0.23	0.70	0.64
3 out of 7	0.47	0.87	0.88	0.46
4 out of 7	0.67	0.77	0.85	0.55
5 out of 7	0.85	0.48	0.76	0.63
4 out of 8	0.55	0.84	0.87	0.49
5 out of 8	0.73	0.68	0.81	0.57
6 out of 8	0.87	0.32	0.71	0.56

*3 Odorant Subgroup (Bubblegum, Chocolate, and Smoke).*

*4 Odorant Subgroup (3 odorants + Wintergreen).*

*5 Odorant Subgroup (4 odorants + Paint thinner).*

*6 Odorant Subgroup (5 odorants + Orange).*

*7 Odorant Subgroup (6 odorants + Strawberry).*

*8 Odorant Subgroup (7 odorants + Grass). Bold values indicates final odorant selection cutoff*.

Cognition can be impaired in PD, especially in people with freezing of gait. In our cohort, as in general practice, MoCA scores were lower in freezers. We therefore performed subgroup analysis on two subgroups of our enrolled participants based on their MoCA scores to explore the independence of our results to cognitive function. We selected and analyzed separately participants with MoCA scores ≥18 (31 non-freezers, 54 freezers), and also analyzed separately participants with MoCA scores ≥25 (24 non-freezers, 33 freezers). In both subgroups, bubblegum, chocolate, and smoke were amongst the top six odors that were most frequently incorrectly identified by freezers compared to non-freezers. The categorization statistics were similar for the 3-odorants (bubblegum, chocolate, and smoke; cut-off ≤ 2 odors identified), in both subgroups (MoCA ≥ 18: 80% sensitivity, 52% specificity, 74% PPV, and 59% NPV; MoCA ≥ 25: 79% sensitivity, 63% specificity, 74% PPV, and 68% NPV) when compared to the full cohort.

Categorization of the severity of hyposmia based on the total UPSIT score has different ranges in males and females. We therefore also calculated predictive statistics for the participants from the two sexes separately with similar results (Supplementary Table 2).

The 3-odorant scores were not correlated with age at the time of visit (non-freezers: −0.017, *p* = 0.928; freezers: −0.093, *p* = 0.480), disease duration (non-freezers: 0.034, *p* = 0.855; freezers: −0.108, *p* = 0.411), motor UPDRS (non-freezers: −0.051, *p* = 0.784; freezers: −0.181, *p* = 0.167), total UPDRS (non-freezers: −0.027, *p* = 0.885; freezers: −0.199, *p* = 0.128) or MoCA scores (non-freezers: 0.350, *p* = 0.054; freezers: 0.165, *p* = 0.208). The independence of our 3-odorant score on disease severity (motor UPDRS and total UPDRS scores) and disease duration was further assessed by plotting these parameters against our 3-odorant scores for each individual using scatter plots and performing linear regression analysis (Supplementary Figure 1). The scatter of values in the different groups was broad and linear regression fits were poor (all *R*^2^ < 0.05) suggesting independence of odorant scores on measures of disease severity in our cohort.

Potential medical conditions, medications (31), and exposures that can potentially impact olfaction were tabulated for all participants (Table 1). Levodopa was not included in this determination as almost all PD participants were on levodopa. The frequency of potential medical conditions were low (all 10% or less) and were not significantly different between groups. For medications, only amlodipine use was more common in non-freezers (five non-freezers), although only seven participants were on this common medication. We also performed group wise analysis stratifying our cohort in different ways. There were no differences in UPSIT scores in those who used or did not use a nasal spray (mean difference = 0.3 ± 3.1 on nasal spray, *p* = 0.926), with or without a past medical co-morbidity (mean difference = 0.5 ± 1.7 with medical co-morbidity, *p* = 0.783), on or off a potential olfactory modulating medication (mean difference = −0.7 ± 0.6 on a medication, *p* = 0.732) or with any one of the above potential confounders (mean difference = 0.6 ± 1.7 with a potential confounder, *p* = 0.711). There were also no differences in our final 3-odorant scores in those who used or did not use a nasal spray (mean difference = −0.2 ± 0.3 on nasal spray, p=0.512), with or without a past medical co-morbidity (mean difference = −0.05 ± 0.18 with medical co-morbidity, *p* = 0.794), on or off a potential olfactory modulating medication (mean difference = 0.02 ± 0.16 with medical co-morbidity, *p* = 0.895), or with any one of the above potential confounders (mean difference = −0.04 ± 0.16 with a potential confounder, *p* = 0.791).

The ability of the B-SIT (10) and the B-SIT B (32), both subscores of the total UPSIT, for categorizing freezers and non-freezers was also calculated for our cohort. At best, these were not as good as our 3-odorant scores for categorizing freezers and non-freezers (B-SIT, ≤ 7/12 odorants identified, sensitivity 70%, specificity 62%, PPV = 79%, NPV = 50%; B-SIT B ≤ 6/12 odorants identified, sensitivity 67%, specificity 52%, PPV = 72%, NPV = 44%).

## Discussion

In this study, we report for the first time that people with PD with or without freezing of gait have differential ability to identify odors on the validated University of Pennsylvania Smell Identification Test (UPSIT). We also found that identification of 2 or fewer out of 3 odors (bubblegum, chocolate, and smoke) had a 77% sensitivity and 61% specificity for categorizing freezers and non-freezers. While these numbers do not suggest that this test could be used in and of itself to differentiate potential freezers from non-freezers, it should be used as a component in future development of predictive algorithms. That being said, our panel showed comparable sensitivity and specificity to previously published shortened tests proposed to help differentiate PD from controls [see (9) for review table]. These include Double et al. (11) (4/5 item cutoff, sensitivity 79%, specificity 58%), Bohnen et al. (23) (2/3 item cutoff, sensitivity 73%, specificity 70%), the Pocket Smell Test (33) (3/3 item cutoff, sensitivity 78%, specificity 54%), and Hawkes et al. (32) (2/2 item cutoff, sensitivity 79%, specificity 50%). Of the three odors we isolated, only bubblegum has not previously been utilized to differentiate PD from controls.

In PD, it has been suggested by the Braak hypothesis that Lewy body propagation may start in the olfactory bulb (and gut) (4). The olfactory bulb has a complex organization making it difficult to attribute odors to particular olfactory epithelia (34, 35), and Lewy bodies have been reported more commonly in the anterior olfactory nucleus (36). Olfactory neurons (including dopaminergic ones) are also replenished by neurogenesis throughout life (37), and in rats, transection of the nigrostriatal dopaminergic pathways led to increased neurogenesis in the olfactory bulb (38). Odor recognition, requires higher levels of processing, and these cortical and hippocampal areas, per the Braak hypothesis are not affected till later stages of disease (4). PD phenotypes have also been reported to have different UPSIT scores (39), but not specific olfactory phenotypes as in our study, and a normosmic PD phenotype with a more benign course has also been reported (2). It is possible that olfactory epithelia are differentially affected in different individuals, propagate pathology along different pathways [as per the prion-like hypothesis of spread (40)], leading to regional variation, or even differential somatosensory/motor/limbic pathway involvement, and thereby different phenotypic expression, such as earlier or later onset of gait freezing. Dysfunction in cholinergic and dopaminergic pathways has been proposed to subserve dysfunctional olfaction (34).

Currently little is known about the pathophysiology of freezing of gait. A study of pathologically confirmed Parkinson's disease patients suggested that faster progression and more severe gait freezing was associated with greater severity in cortical Lewy bodies on autopsy (24). Another study showed no relation between locus coeruleus Lewy Body pathology and UPDRS III gait scores (41). As suggested above for olfactory dysfunction, deficits in the cholinergic pathway have been proposed as a mechanism leading to freezing of gait, with neocortical cholinergic denervation (42) and decreased vesicular acetylcholine transporter binding in the striatum, temporal and mesofrontal limbic regions (43). Cerebellar pathways have been implicated in a number of imaging studies. An analysis of lesion-based freezing in 14 patients, reported that 13/14 different lesions were functionally connected to the dorsal medial cerebellum (44). One study (45) reported increased within cerebellar connectivity and decreased connectivity between cerebellar deep nuclei and frontal cortex. Imaging during imagined gait has also suggested involvement of the cerebellar locomotor region (46). Connectivity between the left globus pallidus and left somatosensory cortex as well as between two areas in the insular/vestibular and default networks were also related to freezing severity (47).

In our cohort, the 3-odorant score did not show significant correlation with age, disease duration, UPDRS scores, or MoCA scores in either the freezers or non-freezers. The predictive statistics were also similar between males and females when run independently. This suggests that our 3-odor score could be applied independent of these features. However, in a prior study in the PD population, not accounting for freezing status, the total UPSIT scores was reported to be correlated with motor UPDRS scores (16). Grading severity from normosmia to complete anosmia is also accomplished using age and sex based tables provided in the UPSIT administration manual.

There are potential limitations to our study. In our cohort, lower MoCA scores were present in the freezers. However, when we used different subgroups, the group with MoCA scores ≥25 had similar predictive statistics on the 3-odorant scores as the whole cohort, and there was no correlation of the 3-odorant scores with MoCA scores, suggesting that cognitive dysfunction did not drive our results. A previous study also did not find a relation between MMSE scores and odor identification using Sniffin sticks (48). Sniffing impairment (as a motor process) has also been reported in PD (49). As we did not measure air-flow-rate, we could not say whether differential smell identification in freezers was secondary to differential sniffing rates. However, one would not expect impaired flow-rate to selectively impact certain odorants unless there was a structural distribution of epithelia for different chemokines that was impacted by sniffing rates. There were also many odors that both freezers and non-freezers had equal difficulty smelling. We did not specifically exclude for medical conditions or medications that can impact odorant identification, but our analysis suggested these factors did not significantly impact our results. Freezing of gait also results in more severe motor disease and therefore UPDRS scores and disease duration were not well-matched between our PD cohorts. However, analysis of the cohorts showed no clear distributional differences of our 3-odorant score to suggest a dependence on disease duration or motor or total UPDRS scores in our cohort.

In summary, we describe a 3-odorant subset of the UPSIT that was able to categorize PD patients with and without freezing of gait, with similar sensitivity and specificity to prior reported short battery tests used to help distinguish aging controls from those with PD. This could suggest that selective dysfunction of the olfactory bulb at disease onset, with subsequent propagation in accordance with the Braak hypothesis, could lead to specific sub-phenotypes of PD. Future longitudinal studies will help determine if differential smell identification could be used to determine PD patients prone to later development of freezing.

## Data Availability Statement

The study is currently bound by institutional review board in regards to data sharing. A de-identified dataset can be made available upon request to the corresponding author.

## Ethics Statement

The studies involving human participants were reviewed and approved by University of Arkansas for Medical Sciences Institutional Review Board. The patients/participants provided their written informed consent to participate in this study.

## Author Contributions

TV and RD were involved in the conception and design of the work. AG and LP were involved in the acquisition and analysis of the data. TV and AG were responsible for statistical analysis and drafting of the manuscript, while all authors were involved in review and revision of the manuscript and acceptance of the final submitted form. All authors are personally accountable for their contributions and the integrity of the work submitted.

## Conflict of Interest

The authors declare that the research was conducted in the absence of any commercial or financial relationships that could be construed as a potential conflict of interest.

## Publisher's Note

All claims expressed in this article are solely those of the authors and do not necessarily represent those of their affiliated organizations, or those of the publisher, the editors and the reviewers. Any product that may be evaluated in this article, or claim that may be made by its manufacturer, is not guaranteed or endorsed by the publisher.
