# Supplementary material for: Olfactory Deficits in the Freezing of Gait Phenotype of Parkinson's Disease
Source: Front Neurol. 2021 Aug 12;12:656379. doi: 10.3389/fneur.2021.656379 (PMC8397477; doi:10.3389/fneur.2021.656379)
Supplement: Supplementary file 1 [file Data_Sheet_1.PDF]

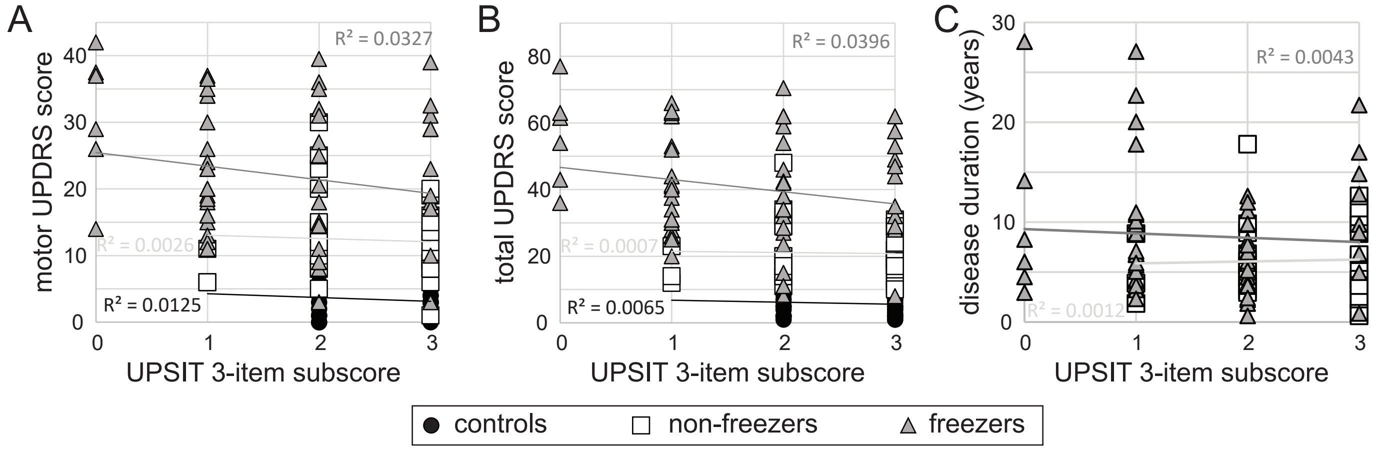

Supplementary Figure 1: Scatter plots showing **(A)** motor UPDRS, **(B)** total UPDRS, and **(C)** disease duration as a function of 3-odorant scores for controls (black circles), PD without freezing (white squares) and and PD with freezing (gray triangles). Best fit lines and regression fit (R^2) values for the different groups are show no significant relationship for controls (black text), PD without freezing (light gray text) and PD with freezing (dark gray text).
