# Supplementary material for: Olfactory Deficits in the Freezing of Gait Phenotype of Parkinson's Disease
Source: Front Neurol. 2021 Aug 12;12:656379. doi: 10.3389/fneur.2021.656379 (PMC8397477; doi:10.3389/fneur.2021.656379)
Supplement: Supplementary file 2 [file Table_1.DOCX]

Supplementary Material

# Supplementary Table 1

| **Supplementary Table 1 – Participant Medical & Surgical History** | | | |
| --- | --- | --- | --- |
|  | controls (n=33)  No. (%) | non-freezers (n=31)  No. (%) | freezers (n=60)  No. (%) |
| **Past Medical History** |  |  |  |
| Neurologic diseases: |  |  |  |
| Ischemic TIA/Stroke | 0 (0%) | 0 (0%) | 1 (2%) |
| Hemorrhagic stroke | 0 (0%) | 0 (0%) | 0 (0%) |
| Seizures or Epilepsy | 0 (0%) | 0 (0%) | 0 (0%) |
| Memory loss or Dementia | 0 (0%) | 1 (3%) | 6 (10%) |
| Migraine headaches | 3 (9%) | 3 (10%) | 4 (7%) |
| Polio | 0 (0%) | 0 (0%) | 1 (2%) |
| Guillain Barre Disease | 0 (0%) | 0 (0%) | 1 (2%) |
| Fibromyalgia | 1 (3%) | 0 (0%) | 1 (2%) |
| Myelomalacia | 0 (0%) | 0 (0%) | 1 (2%) |
| Psychiatric diseases: |  |  |  |
| Depression and/or Anxiety^#^ | 2 (6%) | 5 (16%) | 27 (45%) |
| Depression only^#^ | 0 (0%) | 5 (16%) | 20 (33%) |
| Anxiety only^#^ | 0 (0%) | 4 (13%) | 15 (25%) |
| Thyroid disease | 2 (6%) | 8 (26%) | 8 (13%) |
| Cardiovascular diseases (hyperlipidemia, hypercholesterolemia, heart dz, circulation issues, cardiogenic syncope, valve issues, Raynaud's) | 6 (18%) | 4 (13%) | 13 (22%) |
| Heart disease | 2 (6%) | 9 (29%) | 12 (20%) |
| Orthostasis^#^ | 0 (0%) | 4 (13%) | 10 (17%) |
| Syncope | 0 (0%) | 0 (0%) | 3 (5%) |
| Hypotension | 0 (0%) | 0 (0%) | 4 (7%) |
| Hypertension | 8 (24%) | 14 (45%) | 25 (42%) |
| Hyperlipidemia^#^ | 0 (0%) | 5 (16%) | 10 (17%) |
| Anemia or blood disorder | 2 (6%) | 1 (3%) | 2 (3%) |
| Diabetes | 3 (9%) | 2 (6%) | 8 (13%) |
| Pulmonary diseases (OSA, asthma)^#^ | 3 (9%) | 0 (0%) | 14 (23%) |
| Gastrointestinal diseases (GERD, Gallbladder, Ulcers, constipation, diarrhea, bleeding)^#^ | 3 (3%) | 15 (48%) | 28 (47%) |
| Urogenital diseases (incontinence, nephrolithiasis, BPH, erections, bladder, prostrate, or kidneys)^#^ | 3 (9%) | 17 (55%) | 46 (77%) |
| Infectious diseases: (Pneumonia, meningitis, venereal disease, AIDS, polio, shingles, encephalitis, shingles, sinusitis) | 3 (9%) | 4 (13%) | 6 (10%) |
| Cancer | 5 (15%) | 6 (19%) | 11 (18%) |
| Breast cancer | 0 (0%) | 0 (0%) | 1 (2%) |
| Prostate cancer | 0 (0%) | 1 (3%) | 2 (3%) |
| Colon cancer | 0 (0%) | 0 (0%) | 1 (2%) |
| Melanoma | 0 (0%) | 0 (0%) | 4 (7%) |
| Skin cancer (other than melanoma) | 2 (6%) | 4 (13%) | 2 (3%) |
| Other (Throat and Esophageal) | 1 (3%) | 1 (3%) | 1 (2%) |
| Musculoskeletal diseases |  |  |  |
| Arthritis | 1 (3%) | 1 (3%) | 6 (10%) |
| Osteoporosis | 1 (3%) | 1 (3%) | 1 (2%) |
| Chronic back pain^#^ | 0 (0%) | 0 (0%) | 9 (15%) |
| Fibula fracture | 0 (0%) | 0 (0%) | 1 (2%) |
| Skull fracture | 0 (0%) | 0 (0%) | 1 (2%) |
| Gout | 0 (0%) | 0 (0%) | 1 (2%) |
|  |  |  |  |
| **Past Surgical History** |  |  |  |
| Neurosurgical/orthopedic: |  |  |  |
| Knee replacement surgery | 2 (6%) | 1 (3%) | 4 (7%) |
| Cervical fusion | 0 (0%) | 0 (0%) | 1 (2%) |
| Lumbar disc surgery | 0 (0%) | 0 (0%) | 1 (2%) |
| Bilateral foot surgery | 0 (0%) | 0 (0%) | 1 (2%) |
| Other (appendectomy, tonsillectomy, Carpal Tunnel Release, hernia repair, etc.) | 13 (39%) | 8 (26%) | 18 (30%) |
| ^#^Statistically significant difference (P<0.05) in distribution on chi-square test | | | |

# Supplementary Table 2

| **Supplementary Table 1 – Predictive statistics for males and females including cognitive subgroups** | | | | | | | | |
| --- | --- | --- | --- | --- | --- | --- | --- | --- |
|  | **Females** | | | | **Males** | | | |
| **Number correct** | **Sensitivity** | **Specificity** | **PPV** | **NPV** | **Sensitivity** | **Specificity** | **PPV** | **NPV** |
| Full cohort: (females: 26 freezers, 11 non-freezers, males: 34 freezers, 20 non-freezers) | | | | | | | | |
| 1 out of 3 | 0.50 | 0.82 | 0.87 | 0.41 | 0.47 | 0.95 | 0.94 | 0.51 |
| **2 out of 3** | **0.85** | **0.45** | **0.79** | **0.56** | **0.79** | **0.55** | **0.75** | **0.61** |
| 3 out of 3 | 1.00 | 0.00 | 0.71 | 0.00 | 1.00 | 0.00 | 0.63 | 0.00 |
| MoCA ≥18 subgroup: (females: 24 freezers, 11 non-freezers, males: 30 freezers, 20 non-freezers) | | | | | | | | |
| 1 out of 3 | 0.46 | 0.82 | 0.85 | 0.41 | 0.43 | 0.95 | 0.93 | 0.53 |
| **2 out of 3** | **0.83** | **0.45** | **0.77** | **0.56** | **0.77** | **0.55** | **0.72** | **0.61** |
| 3 out of 3 | 1.00 | 0.00 | 0.69 | 0.00 | 1.00 | 0.00 | 0.60 | 1.00 |
| MoCA≥25 subgroup: (females: 17 freezers, 8 non-freezers, males: 16 freezers, 16 non-freezers) | | | | | | | | |
| 1 out of 3 | 0.47 | 0.88 | 0.89 | 0.44 | 0.44 | 1.00 | 1.00 | 0.64 |
| **2 out of 3** | **0.76** | **0.50** | **0.76** | **0.50** | **0.81** | **0.69** | **0.72** | **0.79** |
| 3 out of 3 | 1.00 | 0.00 | 0.68 | 0.00 | 1.00 | 0.00 | 0.50 | 0.00 |
|  | 3 Odorant Subgroup (Bubblegum, Chocolate, and Smoke) | | | | 3 Odorant Subgroup (Bubblegum, Chocolate, and Smoke) | | | |
